# Assessment of omeprazole and famotidine effects on the pharmacokinetics of tacrolimus in patients following kidney transplant–randomized controlled trial

**DOI:** 10.3389/fphar.2024.1352323

**Published:** 2024-04-04

**Authors:** Miłosz Miedziaszczyk, Marek Karczewski, Tomasz Grabowski, Anna Wolc, Ilona Idasiak-Piechocka

**Affiliations:** ^1^ Department of General and Transplant Surgery, Poznan University of Medical Sciences, Poznan, Poland; ^2^ Department of Inorganic Chemistry, Medical University of Gdansk, Gdansk, Poland; ^3^ Department of Animal Science, Iowa State University, Ames, IA, United States; ^4^ Hy-Line International, Dallas Center, IA, United States

**Keywords:** omeprazole, famotidine, tacrolimus pharmacokinetics, kidney transplant, pharmacotherapy

## Abstract

Tacrolimus is metabolized in the liver with the participation of the CYP3A4 and CYP3A5 enzymes. Proton pump inhibitors are used in kidney transplant patients to prevent duodenal and gastric ulcer disease due to glucocorticoids. Omeprazole, unlike famotidine, is a substrate and inhibitor of the enzymes CYP2C19, CYP3A4, CYP3A5. The aim of this study was to compare the impact of omeprazole and famotidine on the pharmacokinetics of tacrolimus. A randomized, non-blinded study involving 22 stabilized adult kidney transplant patients was conducted. Patients received the standard triple immunosuppression regimen and omeprazole 20 mg (n = 10) or famotidine 20 mg (n = 12). The study material consisted of blood samples in which tacrolimus concentrations were determined using the Chemiluminescent Microparticle Immuno Assay method. A single administration of omeprazole increased tacrolimus concentrations at 2 h (day 2) = 11.90 ± 1.59 ng/mL vs. 2 h (day 1 — no omeprazole administration) = 9.40 ± 0.79 ng/mL (*p* = 0.0443). AUC_0-6_ amounted to 63.07 ± 19.46 ng × h/mL (day 2) vs. 54.23 ± 10.48 ng × h/mL (day 1), (*p* = 0.0295). AUC_2-6_ amounted to 44.32 ± 11.51 ng × h/mL (day 2) vs. 38.68 ± 7.70 ng × h/mL (day 1), (*p* = 0.0130). Conversely, no significant changes in values of pharmacokinetic parameters were observed for famotidine. Omeprazole significantly increases blood exposure of tacrolimus. The administration of famotidine instead of omeprazole seems safer for patients following kidney transplantation.

**Clinical Trial Registration:** clinicaltrials.gov, identifier NCT05061303

## 1 Introduction

Currently, the majority of immunosuppressive regimens employed after kidney transplantation include tacrolimus (Franco et al., 2019). Tacrolimus is an calcineurin inhibitor used to prevent rejection in allogeneic organ transplant recipients, such as kidney, liver, heart or lungs. It is a substrate for intestinal P-glycoprotein (Marfo et al., 2010). Tacrolimus is metabolized in the liver with the participation of the cytochrome P450 isoform CYP3A4 and CYP3A5 (CYP3A4, CYP3A5) (Floren et al., 1997; Christians et al., 1996), and is characterized by a narrow therapeutic window, dose-dependent toxicity and high inter-individual and intra-individual variability. Tacrolimus is a nephrotoxic drug. Additionally, tacrolimus blood concentrations require regular monitoring during therapy, which is referred to as therapeutic drug monitoring (Budde et al., 2022). It is recommended to maintain tacrolimus C_0_ trough concentrations >7 ng/mL in the first year after kidney transplantation in patients with low immunological risk, treated concomitantly with mycophenolate mofetil and glucocorticoids in combination with basiliximab induction. For recipients with increased immunological risk, higher tacrolimus C_0_ concentrations are recommended: 10–20 ng/mL during the first 2 months, then 5–10 ng/mL. In Poland, induction with basiliximab is not routinely used in kidney transplant recipients with low immunological risk, therefore national recommendations are different (Marfo et al., 2010). The 6-year survival of the kidney graft was over 88% for the tacrolimus C_0_ concentration ranges: 5.0–6.9 ng/mL and 7.0–8.9 ng/mL in the first 3 years after transplantation, 87.5% for ≥9 ng/mL, 86.5% for the range of 4.0–4.9 ng/mL and only 82.6% for C_0_ values <4 ng/mL. The process of chronic rejection is most effectively inhibited when tacrolimus concentrations are maintained within the C_0_ range: 5.0–8.9 ng/mL (Susal and Dohler, 2019). Increased blood concentrations of tacrolimus may be affected by CYP3A4 substrates and inhibitors, resulting in adverse effects of this drug, particularly nephrotoxicity (Miedziaszczyk and Idasiak-Piechocka, 2023). Proton pump inhibitors (PPIs) are administered as a standard treatment method in kidney transplant patients in order to prevent duodenal and gastric ulcer disease due to pro-inflammatory effects of immunosuppressive agents use or infections (Takahashi et al., 2007; Ponticelli and Passerini, 2005; Telkes et al., 2011). Glucocorticoids represent one of the most frequently employed triple immunosuppression regimens following kidney transplantation. One of the PPIs is omeprazole, which is a substrate and inhibitor of the enzymes CYP2C19, CYP3A4, CYP3A5, and simultaneously a P-glycoprotein inhibitor (Moreau et al., 2006; Pauli-Magnus et al., 2001). CYP2C19 enzyme is primarily involved in the metabolism of omeprazole; however, upon saturation or in the event of a mutation in the CYP2C19 gene (in poor metabolizers), CYP3A4/5 emerges as the major enzyme participating in the elimination of omeprazole and, thus, may interact with tacrolimus (Zhao WFM. et al., 2012; Hosohata et al., 2009; Maguire et al., 2012; Boso et al., 2013). Therefore, it seems vital to administer medications with a similar gastro-protective effect, such as famotidine - an antagonist of H2 receptors that, contrary to omeprazole, are not substrates or inhibitors of the CYP3A4/5 enzyme. There are no studies in the available scientific literature comparing changes in tacrolimus concentrations in patients following kidney transplantation receiving concomitant omeprazole vs. receiving famotidine instead of omeprazole. Our study is the first to present results comparing the above-mentioned research groups. The potential impact of omeprazole on the pharmacokinetics of tacrolimus (Franco et al., 2019; Floren et al., 1997; Christians et al., 1996; Budde et al., 2022; Takahashi et al., 2007; Moreau et al., 2006; Zhao W. et al., 2012; Hosohata et al., 2009; Maguire et al., 2012; Boso et al., 2013; Sugimoto and Furuta, 2012; Dehbozorgi et al., 2018; Pascual et al., 2005; Peloso et al., 2014), may result in fluctuations in the blood concentrations, drug toxicity and, consequently, lead to the development of chronic rejection of the transplanted kidney. It is important to investigate omeprazole-tacrolimus interaction, as well as to explore neutral therapeutic substances to prevent gastrointestinal complications. The use of famotidine instead of omeprazole may prove a more beneficial and safer treatment option for renal transplant patients. Bearing in mind these observations, the aim of the study was to compare the effects of omeprazole and famotidine on the pharmacokinetics of tacrolimus in patients following kidney transplant.

## 2 Materials and methods

**FIGURE 1:**
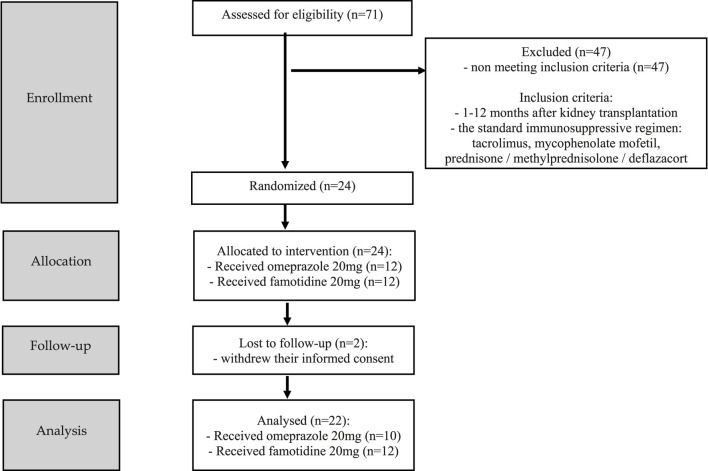
CONSORT flow chart.

**FIGURE 2:**
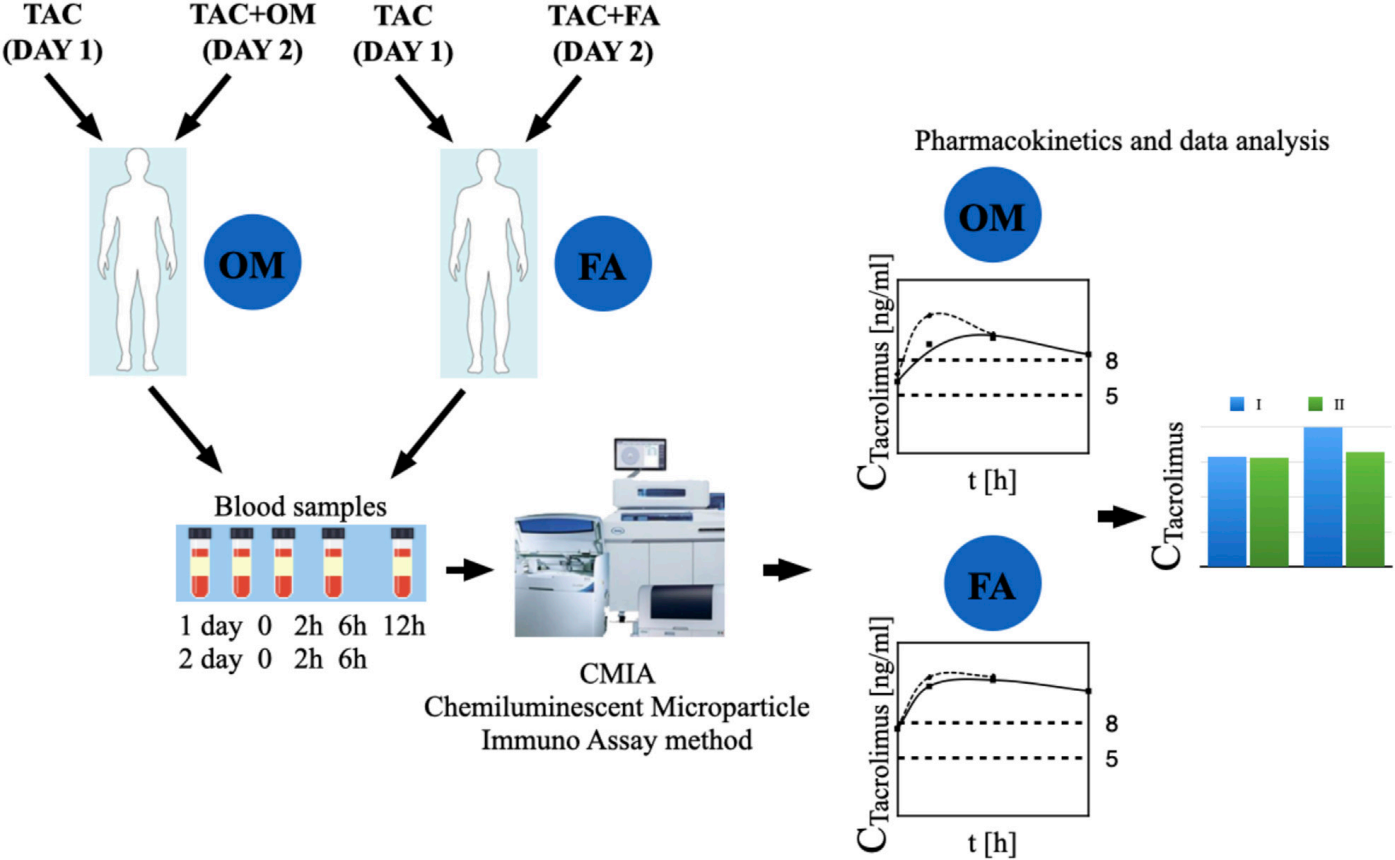
The graphic representation of the study methodology. TAC - patients receiving tacrolimus alone on the first day of the study, TAC + OM—patients receiving tacrolimus together with omeprazole on the second day of the study, TAC + FA—patients receiving tacrolimus together with omeprazole on the second day of the study.

The study, randomized, non-blinded, comprised 24 stabilized adult patients between 1–12 months after kidney transplantation (NCT05061303), who received a kidney from a deceased donor. Inclusion criteria were time since kidney transplantation (1–12 months after kidney transplantation) and standard immunosuppressive regimen (tacrolimus, mycophenolate mofetil, prednisone/methylprednisolone/deflazacort). In this group, 2 individuals withdrew their informed consent and were excluded from study. Each randomized patient was admitted to the Department of Nephrology, Transplantology and Internal Diseases of Poznan University of Medical Sciences for 2 days (48 h). The study used the simple randomization method. One of the authors of the study (no the principal researcher) generated a randomized sequence for assigning patients to groups. The random component (computer-generated random numbers) was used in the sequence generation process. Figure 1 presented CONSORT flow chart. No participant changed the group during the study. Standard laboratory tests (creatinine, estimated glomerular filtration rate (eGFR), complete blood count, proteinuria, erythrocyturia) were performed during the first 2 days of participation in the study. In addition, the glomerular filtration rate (GFR) was calculated for each patient using The Chronic Kidney Disease Epidemiology Collaboration (CKD-EPI) creatinine equation to estimate GFR. The baseline characteristics of the study groups are presented in Table 1. Patients received orally the standard, most commonly administered triple immunosuppressive regimen: tacrolimus, mycophenolate mofetil, prednisone/methylprednisolone/deflazacort and orally: omeprazole 20 mg (group I), or famotidine 20 mg (group II) depending on the random classification to the groups. Patients received tacrolimus once daily in the form of tacrolimus monohydrate prolonged-release tablets (Envarsus^®^, Chiesi Farmaceutici), registration numbers: EU/1/14/935/00, EU/1/14/935/004, EU/1/14/935/006, EU/1/14/935/007 or in the form of tacrolimus monohydrate prolonged-release hard capsules (Advagraf^®^, Astellas Pharma Europe), registration numbers: EU/1/07/387/001, EU/1/07/387/003, EU/1/07/387/011, EU/1/07/387/007. Notably, patients were continuously treated with tacrolimus prior to entering the study. All patients before the start of the study achieved tacrolimus steady state. The material for the study involved blood samples in which tacrolimus concentrations were determined at the following time points: 0 h, 2 h, 6 h, 12 h after drug administration, when no omeprazole/famotidine was administered, and then the following day after receiving gastro-protective medications at the same time points, except for 12 h time point. Since the elimination half-life of omeprazole in the body is shorter than an hour, no significant effect of omeprazole on tacrolimus metabolism was expected after a few hours, therefore, tacrolimus concentrations were not assessed at the 12 h time point during the second day of hospitalization to avoid exposed patients to additional blood donation (Summary of Product Characteristics ofa) (Summary of Product Characteristics ofb). The results were used to obtain the values of pharmacokinetic parameters and statistical evaluation. A graphical representation of the research methodology is presented in Figure 2. No study-related adverse events were reported by patients, physicians, medical staff, or others during the study.

**TABLE 1:** The characteristics of the study groups. The values are provided as the number of patients (n) or as mean ± SD.

Characteristic parameter	Group I	Group II	Shapiro-Wilk test (*p*-value)	95% Cl	t-test for non-dependent groups (*p*-value)
Gender (n; men/women)	5/5	10/2	-	-	-
Age (years)	41 ± 15	50 ± 9	*p* = 0.4999	−2.3895 to 19.7228	*p* = 0.1177
Body Mass Index (kg/m^2^)	23.30 ± 3.33	26.65 ± 5.08	*p* = 0.2699	−0.5621 to 7.2561	*p* = 0.0893
systolic/diastolic blood pressure (SBP/DBP) (mmHg) arterial hypertension	141.30 ± 21.50/90.10 ± 11.78 (n = 6)	146.73 ± 20.18/92.45 ± 13.58 (n = 6)	SBP *p* = 0.5590	SBP -13.6108 to 24.4654	SBP *p* = 0.5578
			DBP *p* = 0.9429	DBP -9.3148 to 14.0238	DBP *p* = 0.6775
Creatinine concentration (mg/dL)	Q1: 1.12	Q1: 1.37	**p = 0.0331**	-	*p* = 0.0503^1^
	Q2: 1.31	Q2: 1.50			
	Q3: 1.38	Q3: 1.87			
Glomerular Filtration Rate [The Chronic Kidney Disease - Epidemiology Collaboration] (mL/min/1,73 m^2)^	67.80 ± 25.87	51.75 ± 14.11	*p* = 0.4628	−34.1503 to 2.0503	*p* = 0.0792
Tacrolimus daily dose (mg/kg)	0.0611 ± 0.0381	0.0376 ± 0.0162	*p* = 0.0925	−0.0516 to 0.0047	*p* = 0.0948^2^
Proteinuria (n)	1	5	-	-	-
Erythrocyturia (n)	0	2	-	-	-
Cytomegalovirus (n)	3	1	-	-	-
BK virus (n)	0	1	-	-	-
Anaemia (n)	1	3	-	-	-
the mean C_0_/D ratio (ng/mL*1/mg)	2.02 ± 1.14	2.49 ± 0.76	*p* = 0.4425	−0.2471 to 1.1338	*p* = 0.1694
poor metabolizers (n)	5	10	-	-	-
intermediate metabolizers (n)	3	2	-	-	-
ultra-rapid metabolizers (n)	2	0	-	-	-

1—the Mann-Whitney U test.

2—the Welch’s t-test.

Values in bold are statistically significant.

In order to establish the tacrolimus metabolism rate in the patients, the blood concentration normalized by the dose (C/D ratio) was calculated. The C_0_/D ratio can be calculated by dividing the tacrolimus pre-dose concentration (C_0_) by the corresponding daily tacrolimus dose (D). In the presented study, the authors used the scale suggested by Thölking G. et al. If the C/D ratio was 1.05–1.54 ng/mL*1/mg, the patients were classified as intermediate metabolizers. The fast metabolizers group included patients with a C/D ratio of <1.05 ng/mL*1/mg, whereas if the aforementioned ratio was ≥1.55 ng/mL*1/mg, the participants were described as slow metabolizers (Tholking et al., 2014). The results are presented in Table 1.

### 2.1 Chemiluminescent microparticle immuno assay (CMIA)

Tacrolimus concentrations were determined using CMIA, by means of Alinity i analyser (Mei et al., 2018). Samples were stored frozen (−10°C or below) for no longer than 6 months. Each sample was thoroughly mixed prior to determining concentration, and 200 μL of each sample was pipetted into a centrifuge tube. Subsequently, the dispenser was filled with the appropriate volume of ARCHITECT Tacrolimus Whole Blood Precipitation Reagent, followed by the removal of air bubbles from the dispenser. 200 μL of ARCHITECT Tacrolimus Whole Blood Precipitation Reagent was added to the contents of the first centrifuge tube. Each individual tube was capped, mixed immediately after adding the reagent and centrifuged for 5–10 s. The tubes were then centrifuged again for 4 min. Each tube was uncapped and the supernatant was decanted (poured off) into a Graft Pre-Treatment Tube. They were then vortexed for 5–10 s and assayed for the concentrations of tacrolimus.

Calibration was performed in duplicate samples by testing Calibrators A, B, C, D, E and F. Control samples were prepared by mixing 150 μL of the patient sample and 150 μL of ARCHITECT Tacrolimus Calibrator A. One sample for each control level was tested once every 24 h as a quality control test. The analyst evaluating tacrolimus concentrations had no group assignment information.

### 2.2 Pharmacokinetic evaluation

Pharmacokinetic analysis of tacrolimus concentrations were determined by non-compartmental analysis. Phoenix WinNonlin 8.1 (Certara LC) software was used to calculate the pharmacokinetic parameters. The following parameters were calculated: AUC_0-6_—a fraction of the area under the concentration-time curve between 0 h and 6 h; AUC*_0–6_—a fraction of the area under the concentration-time curve between 0 h and 6 h normalized by the dose; AUC_2-6_—a fraction of the area under the concentration-time curve between 2 h and 6 h; AUC*_2–6_—a fraction of the area under the concentration-time curve between 2 h and 6 h normalized by the dose; C_max_–the peak concentrations in the first and second day.

### 2.3 Statistical analysis

Statistical analysis was performed separately in group I and separately in group II. The values of pharmacokinetic parameters were compared for tacrolimus concentrations without the administration of omeprazole (group I) or famotidine (group II) vs. together with omeprazole or famotidine. The Shapiro-Wilk test was used to verify whether the results of differences in pairs of pharmacokinetic parameters were normally distributed. For normal distribution variables (*p* > 0.05), the paired Student’s t-test was applied to estimate the significance of differences between the two analysed groups. The parameters which were significantly different from the normal distribution (*p* < 0.05) were analysed using the paired Wilcoxon signed-rank test.

The statistical analysis of the baseline values of the study groups was also performed. The Shapiro-Wilk test was used to verify whether the results of differences in pairs of pharmacokinetic parameters were normally distributed. For normal distribution variables (*p* > 0.05), the non-paired Student’s t-test was applied to estimate the significance of differences between the two analysed groups. The parameters which were significantly different from the normal distribution (*p* < 0.05) were analysed using the Mann-Whitney U test. For normal distribution variables (*p* > 0.05), the Welch’s t-test was applied to estimate the significance of differences between the two analysed groups for unequal variances. F test was used to compare the variances between study groups.

The analysis was performed using UNIVARIATE procedure of SAS (SAS Institute Inc. 2002–2012. The SAS System for Windows version 9.4. (Cary, NC, USA).

## 3 Results

**FIGURE 3:**
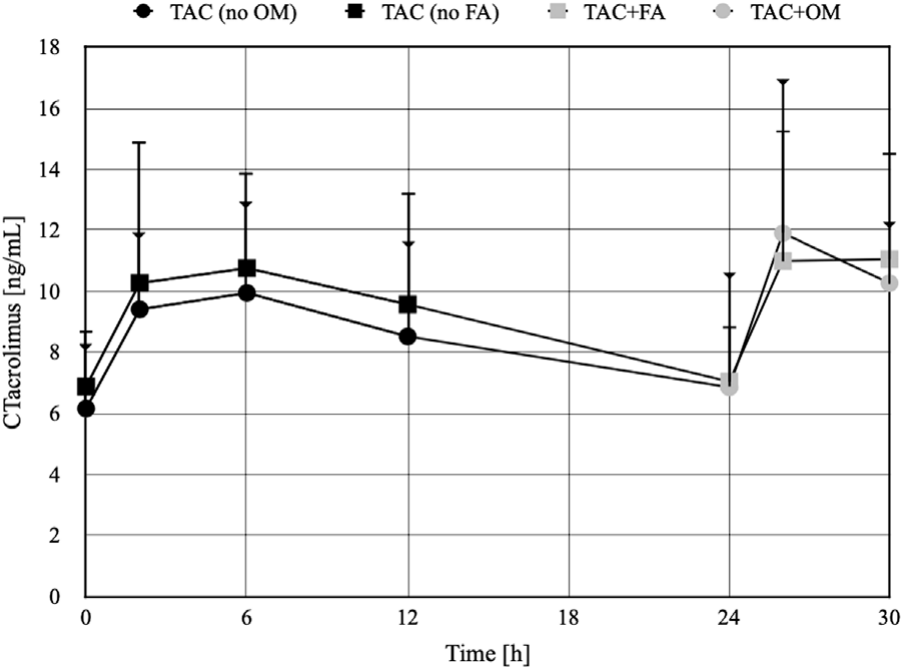
The concentration-time profiles of tacrolimus in patients receiving omeprazole (OM) or famotidine (FA). TAC - patients receiving tacrolimus alone on the first day of the study, TAC + OM - patients receiving tacrolimus together with omeprazole on the second day of the study, TAC + FA—patients receiving tacrolimus together with omeprazole on the second day of the study. The dots on the figure define the mean values, the whiskers represent standard deviation values. The whiskers ending with a triangle refer to the TAC group (no FA) and then TAC + FA. The whiskers ending with a line refer to the TAC group (no OM) and then TAC + OM.

**FIGURE 4:**
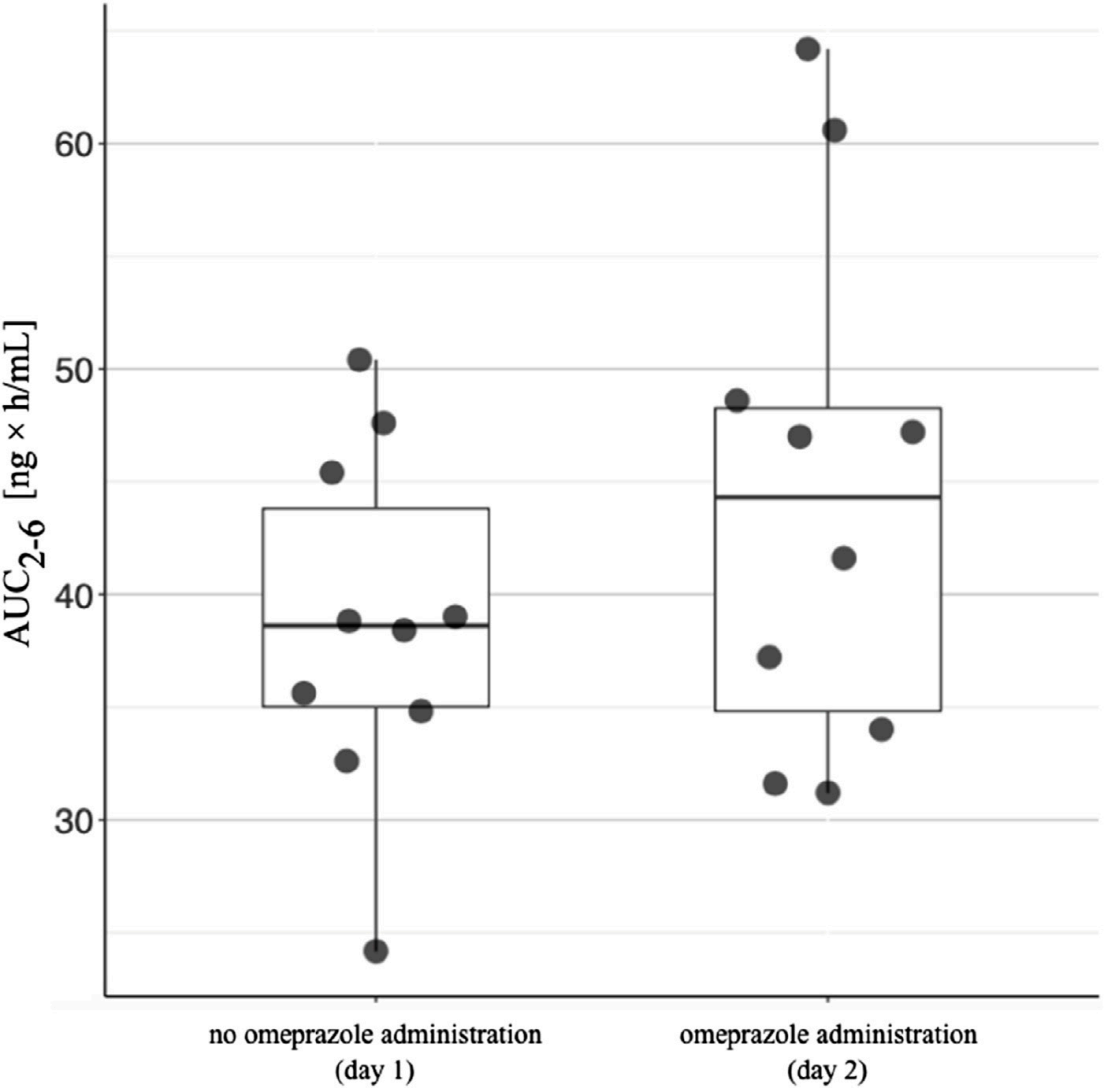
The area under the concentration-time curve between 2 h and 6 h (AUC_2-6_) in patients receiving omeprazole. The AUC_2-6_ values for patients taking tacrolimus without omeprazole are shown on the left. The AUC_2-6_ values in patients taking tacrolimus and omeprazole concomitantly are shown on the right. The lower side of the box is defined by the first quartile, the upper side by the third quartile. The horizontal line inside the box represents the median value. The upper end of the line is the highest value in the group, while the lower end of the line is the lowest value.

The arithmetic mean blood tacrolimus concentrations after a single oral administration to the omeprazole and famotidine groups are shown in Figure 3. The main pharmacokinetic parameters are summarized in Table 2 and Table 3. Omeprazole significantly increased tacrolimus AUC_0-6_ by 16.30% (*p* = 0.0295), AUC_0-6_ normalized by the dose by 12.88% (*p* = 0.0300) and AUC_2-6_ by 14.58% (*p* = 0.0130) (Figure 4), AUC_2-6_ normalized by the dose by 12.74% (*p* = 0.0109). In order to assess whether the difference in the daily dose of tacrolimus between the group receiving omeprazole and the group receiving famotidine affects the obtained study results, dose normalization was used. The obtained results do not lead to different conclusions than those without dose normalization. After administration of omeprazole and tacrolimus, the C_2h_ value increased by 26.60% (*p* = 0.0443). There were no significant differences after administration of omeprazole compared to the previous day when this drug was not administered in the following pharmacokinetic parameters of tacrolimus: C_max_ (*p* = 0.0955), C_0_ (*p* = 0.5876), C_6_ (*p* = 0.6409). Famotidine administration did not result in statistically significant pharmacokinetic parameters: C_max_ (*p* = 0.7199), C_0_ (*p* = 0.3394), C_2_ (*p* = 0.4344), C_6_ (*p* = 0.7374), AUC_2-6_ (*p* = 0.3910), AUC_2-6_ normalized by the dose (*p* = 0.3146), AUC_0-6_ (*p* = 0.3277), AUC_0-6_ normalized by the dose (*p* = 0.2438).

**TABLE 2:** The pharmacokinetic parameters of tacrolimus without and with coadministration of omeprazole 20 mg (OM).

Pharmacokinetic parameters	no OM (day 1)	OM (day 2)	Shapiro-Wilk test (*p*-value)	95% Cl	t-test for dependent groups (*p*-value)
C_max_ (ng/mL)	11.22 ± 2.34	13.07 ± 4.30	*p* = 0.3013	−0.3973 to −4.0973	0.0955
C_0_ (ng/mL)	6.15 ± 2.12	6.85 ± 3.76	*p* = 0.0778	−2.1158 to 3.5158	0.5876
C_2_ (ng/mL)	9.40 ± 2.50	11.90 ± 5.03	*p* = 0.7298	0.0786 to 4.9214	**0.0443**
C_6_ (ng/mL)	9.94 ± 3.03	10.26 ± 1.99	*p* = 0.4530	−1.1801 to 1.8201	0.6409
AUC_0-6_ (ng × h/mL)	54.23 ± 10.48	63.07 ± 19.46	*p* = 0.5495	−15.8264 to 27.8264	**0.0295**
AUC^a^_0–6_	1158.^a^5 ± 556.93	1308.01 ± 595.19	*p* = 0.5489	18.0754 to 280.3968	**0.0300**
((ng × h/mL)/(mg/kg))					
AUC_2-6_	38.68 ± 7.70	44.32 ± 11.51	*p* = 0.7501	1.5050 to 9.7750	**0.0130**
(ng × h/mL)					
AUC^a^_2–6_	832.98 ± 415.81	939.13 ± 453.89	*p* = 0.8026	30.9855 to 181.3068	**0.0109**
((ng × h/mL)/(mg/kg))					

^a^ the area under the concentration-time curve normalized by the dose.

Values in bold are statistically significant.

**TABLE 3:** The pharmacokinetic parameters of tacrolimus without and with coadministration of famotidine 20 mg (FA).

Pharmacokinetic parameters	no FA (day 1)	FA (day 2)	Shapiro-Wilk test (*p*-value)	95% Cl	t-test for dependent groups (*p*-value)
C_max_ (ng/mL)	12.68 ± 4.39	13.05 ± 4.17	*p* = 0.5547	−1.7022 to 2.3856	0.7199
C_0h_ (ng/mL)	Q1: 5.25	Q1: 5.60	**p = 0.0345**	-	0.3394^
	Q2: 6.50	Q2: 7.30			
	Q3: 8.80	Q3: 7.95			
C_2_ (ng/mL)	10.26 ± 4.64	10.98 ± 4.27	*p* = 0.0927	−1.2420 to 2.6920	0.4344
C_6_ (ng/mL)	10.75 ± 3.16	11.04 ± 3.53	*p* = 0.2040	−1.5748 to 2.1582	0.7374
AUC_0-6_	59.15 ± 16.60	62.07 ± 15.92	*p* = 0.8709	−3.3504 to 9.1838	0.3277
(ng × h/mL)					
AUC^a^_0–6_	1677.84 ± 358.32	1784.71 ± 447.89	*p* = 0.7590	−84.0925 to 297.8647	0.2437
((ng × h/mL)/(mg/kg))					
AUC_2-6_ (ng × h/mL)	42.02 ± 11.54	44.05 ± 11.28	*p* = 0.7966	−2.9789 to 7.0456	0.3910
AUC^a^_2–6_	1196.79 ± 279.50	1268.57 ± 329.31	*p* = 0.9537	−78.1345 to 221.6834	0.3146
((ng × h/mL)/(mg/kg))					

^a^ the area under the concentration-time curve normalized by the dose ^ - the paired Wilcoxon signed-rank test.

Values in bold are statistically significant.

## 4 Discussion

In our study, patients receiving omeprazole showed significantly higher tacrolimus concentrations than the participants without OM therapy (Table 2). Nevertheless, the addition of famotidine to pharmacotherapy instead of omeprazole did not result in any significant changes in tacrolimus blood concentrations. Given that both tacrolimus and omeprazole are metabolised by the CYP3A4 isoform of the cytochrome P450 group, there is a potential for drug-drug interactions through enzyme competition. In the available sources, a case report was found which corresponded to the results obtained in our study.

Takayashi K et al. (Takahashi et al., 2007) presented a case of a Japanese patient (32 years old) following kidney transplantation where a change in the concentration of tacrolimus in the blood was observed after replacing ranitidine with omeprazole. Immunosuppressive treatment included tacrolimus, mycophenolate mofetil, and prednisolone. On the post-transplant day 17, the trough concentration of tacrolimus was 12.5 ng/mL with a daily oral dose of 17 mg. Subsequently, on day 18, orally administered ranitidine was changed to intravenous omeprazole for the management of peptic ulcers. With the initiation of omeprazole treatment, tacrolimus trough levels abruptly increased to 30.0 ng/mL on the post-transplant day 19. Despite tacrolimus dose reductions (from 17 to 9 mg/day), tacrolimus trough levels remained above 15 ng/mL. Furthermore, omeprazole was replaced with oral rabeprazole (10 mg/day) on day 21. After switching to rabeprazole, blood concentrations of tacrolimus were well controlled at approximately 10 ng/mL. Due to the fact that informed consent could not be obtained from this patient, the researchers were unable to access genetic information, and thus it was impossible to determine whether the patient had the CYP2C19 gene mutation (Takahashi et al., 2007).

In another case report, an increase in tacrolimus concentrations was also observed after the administration of omeprazole. Zhao W. et al. (Zhao WFM. et al., 2012) found a more than four-fold increase in tacrolimus concentrations after omeprazole administration in a 17-year-old kidney transplant patient. The immunosuppressive regimen in this case comprised tacrolimus, mycophenolate mofetil and methylprednisolone. The initial dose of tacrolimus was 9 mg twice daily, which resulted in tacrolimus blood trough levels (C_0_) of 19.6 and 29.2 ng/mL postoperatively on days 3 and 4, respectively, and the dose was consequently reduced to 7 mg. On postoperative day 5, omeprazole was administered to alleviate gastric reflux. Despite tacrolimus dose reduction, on postoperative day 6, C0 increased to 92 ng/mL and induced acute nephrotoxicity (serum creatinine 160 mmol/L). Tacrolimus treatment was resumed on postoperative day 7 based on TDM results, whereas omeprazole treatment was discontinued. Subsequently, the nephrotoxicity was reversed (serum creatinine returned to baseline and steadily decreased in the days following surgery). The patient was found to be homozygous for the (*2/*2) CYP2C19 mutation, which resulted in an abnormal splicing site that significantly reduced or inactivated the activity of the encoded CYP2C19 protein, and thus was classified as a poor CYP2C19 metabolizer (Zhao W. et al., 2012).

In our study, genetic testing was not conducted, although the patients were evaluated with regard to their drug metabolism rate according to the C/D ratio, as suggested by Thölking G. et al. Patients were classified as intermediate metabolisers with a C/D ratio between 1.05 and 1.54 ng/mL*1/mg. In turn, if the C/D ratio was <1.05 ng/mL*1/mg, they were described as fast metabolizers, whereas if the said ratio was ≥1.55 ng/mL*1/mg, the participants were referred to as slow metabolizers (Tholking et al., 2014). It is of note that the mean C/D ratio was higher in the famotidine group than in the omeprazole group, which emphasise the relevance of our results regarding the significant changes in tacrolimus concentrations observed within the omeprazole group. CYP2C19 is predominantly involved in the metabolism of omeprazole; however, in the case of the CYP2C19 gene mutation (in poor metabolizers), CYP3A4/5 becomes the main enzyme participating in the elimination of omeprazole. In addition, tacrolimus is also metabolized by CYP3A4/5, which may lead to a competition for CYP3A4/5 enzymes whereby the metabolism of tacrolimus may be insufficient. This, in turn, may result in increased the concentrations of tacrolimus, which may affect the process of chronic renal rejection. In contrast, famotidine is not a substrate of CYP3A4/CYP3A5 and, therefore, does not interact with tacrolimus. In their study involving 75 patients, Boso V. et al. demonstrated that recipients with the CYP2C19*2/*2 genotype presented higher blood concentrations of tacrolimus as compared to those without the mutations during treatment with tacrolimus and omeprazole (Boso et al., 2013). Nevertheless, it should also be noted that Pascual J. et al. in a case-analytical study (n = 51) concluded that the interaction of omeprazole with tacrolimus was of no clinical significance. Despite the potential competition or interaction at the molecular level, the clinical management was not significantly affected in renal allograft recipients. Tacrolimus doses and the level/dose ratio were recorded 7 times: at 3 outpatient appointments prior to omeprazole withdrawal (Pre3/Pre2/Pre1), at the withdrawal appointment (Susp), and at 3 post-withdrawal appointments (Pos1/Pos2/Pos3). The abovementioned appointments were approximately 1 month apart. Pascual et al. observed the slow, progressive decline in tacrolimus concentration throughout the study, and hence concluded that there was no significant drug interaction between omeprazole and tacrolimus (Pascual et al., 2005). Conversely, according to a double-blind, placebo-controlled pilot study (n = 28) by Peloso et al., omeprazole may increase the blood concentration of tacrolimus if administered 2 h before receiving tacrolimus, potentially as a result of intestinal contents alkalization (Peloso et al., 2014). On the basis of the available studies and case reports, it seems reasonable to avoid omeprazole administration in patients receiving tacrolimus, regardless of the CYP2C19 genotype (Maguire et al., 2012). It is worth bearing in mind that triclopidine, fluvoxamine, voriconazole, fluconazole, ketoconazole and fluoxetine represent potent inhibitors of the CYP2C19 enzyme (Yasui-Furukori et al., 2004; Jeong et al., 2009; Harvey and Preskorn, 2001). Therefore, the use of a CYP2C19 inhibitor in renal transplant patients where omeprazole and tacrolimus are administered may result in drug interactions. The inhibition of the CYP2C19 isoform may suppress the metabolism of omeprazole by means of the CYP2C19 enzyme. Furthermore, in order to maintain adequate biotransformation, omeprazole may alter its metabolic pathway to be metabolized by the CYP3A4 enzyme, which may lead to drug interactions with tacrolimus. In addition, the competition for the CYP3A4 enzyme may affect the metabolism of tacrolimus, resulting in increased the concentrations of tacrolimus, which in turn may trigger adverse reactions and, consequently, lead to the development of chronic dysfunction of the transplanted kidney. It should also be noted that one of the side effects of tacrolimus is nephrotoxicity. In contrast, famotidine does not interact with the drug-metabolizing enzyme system associated with cytochrome P450, and therefore is a good candidate to be administered instead of omeprazole (Itagaki et al., 2002). In addition to the role of cytochrome P450 described above, the effect of P-glycoprotein (P-gp) is also important in the interaction under study. The increase in tacrolimus exposure observed in our study when coadministered with omeprazole vs. without omeprazole may be a result of intestinal P-gp inhibition by omeprazole (Pauli-Magnus et al., 2001). During tacrolimus-omeprazole interactions at the level of the ABCB1 transporter, the absorption of tacrolimus increases, which may result in an increase in drug concentration in blood (Zhao WFM. et al., 2012; Peng et al., 2020). Additionally, another mechanism related to lipophilicity/binding to plasma proteins of famotidine (low lipophilicity: XlogP3 = −0.6), omeprazole (high lipophilicity: XLogP3 = 2.2), and tacrolimus (high lipophilicity: XLogP3 = 2.7) may play a key role (National Center for Biotechnology Information, 2005a; National Center for Biotechnology Information, 2005b; National Center for Biotechnology Information, 2005c). Omerpazole (approximately 95%) and tacrolimus (approximately 99% of) highly bound to plasma proteins. It means that both drugs compete with binding sites. In effect, more tacrolimus molecules are in non-bound (free fraction) in the bloodstream. In consequence, higher AUC was observed.

C_0_ is used in most transplant centers for routine therapeutic drug monitoring of tacrolimus (Brunet et al., 2019). In our study, we observed that the AUC_2-6_ value plays a greater role in assessing the occurrence of omeprazole-tacrolimus interactions than the C_0_ or C_2_ value. AUC_2-6_ may be an important pharmacokinetic parameter assessing the occurrence of drug-drug interactions in patients following kidney transplantation taking tacrolimus after a single administration of a specific drug. The results of our study may have clinical significance. This is the first cohort study to evaluate the effect of famotidine on tacrolimus concentrations in patients following kidney transplantation. Famotidine have not effected on the pharmacokinetics of tacrolimus, so it may be an alternative to proton pump inhibitors in the prevention of upper gastrointestinal bleeding in patients after kidney transplantation. Moreover, unlike proton pump inhibitors, famotidine does not have a nephrotoxic effect. Moreover, the observed nephrotoxicity in patients following kidney transplantation may be related to the combined use of omeprazole or the effect of the drug on the concentration of nephrotoxic tacrolimus. A significant limitation of our study is the small group of patients. However, despite such a limited number of subjects, our results were found to be significant. Obtaining statistically significant results considering the number of patients studied highlights the significant difference between the change in tacrolimus concentration after administrating with omeprazole vs. without omeprazole. No such change was observed compared to administrating tacrolimus with famotidine. As a result, after repeated combined administration of both drugs, omeprazole may increase tacrolimus concentrations to toxic levels, which may result in nephrotoxicity of the drug. No such change was observed compared to administration of tacrolimus with famotidine. It is important to investigate the effects of omeprazole and famotidine on tacrolimus concentrations after repeated dosing. Furthermore, the number of tacrolimus concentration measurements in the course of the study (30 h) could have been higher. Yet, due to the pain associated with collecting blood samples, it was impossible to determine these concentrations more frequently. It would also be clinically important to conduct a study assessing the long-term effect of the studied drugs on the pharmacokinetics of tacrolimus, as well as assessing the function a transplanted kidney and markers of the rejection process of a transplanted kidney in the study groups over time.

## 5 Conclusion

Co-administration of omeprazole and tacrolimus in renal transplant patients presents greater difficulties in maintaining tacrolimus concentrations within the therapeutic range than the combination of famotidine and tacrolimus. Moreover, in contrast to famotidine, omeprazole significantly increased blood exposure of tacrolimus. Therefore, the use of famotidine instead of omeprazole seems to be more beneficial and safer for patients following kidney transplantation. Given the results of our study and the widely reported nephrotoxicity of omeprazole, the administration of this medication is not recommended in renal transplant patients.

## Data Availability

The original contributions presented in the study are included in the article/Supplementary Material, further inquiries can be directed to the corresponding author.

## References

[B1] Boso V. Herrero M. J. Bea S. Galiana M. Marrero P. Marques M. R. (2013). Increased hospital stay and allograft dysfunction in renal transplant recipients with Cyp2c19 AA variant in SNP rs4244285. Drug Metab. Dispos. 41 (2), 480–487. 10.1124/dmd.112.047977 23175667

[B2] Brunet M. van Gelder T. Asberg A. Haufroid V. Hesselink D. A. Langman L. (2019). Therapeutic drug monitoring of tacrolimus-personalized therapy: second consensus report. Ther. Drug Monit. 41 (3), 261–307. 10.1097/FTD.0000000000000640 31045868

[B3] Budde K. Rostaing L. Maggiore U. Piotti G. Surace D. Geraci S. (2022). Prolonged-release once-daily formulation of tacrolimus versus standard-of-care tacrolimus in *de novo* kidney transplant patients across Europe. Transpl. Int. 35, 10225. 10.3389/ti.2021.10225 36017158 PMC9397503

[B4] Christians U. Schmidt G. Bader A. Lampen A. Schottmann R. Linck A. (1996). Identification of drugs inhibiting the *in vitro* metabolism of tacrolimus by human liver microsomes. Br. J. Clin. Pharmacol. 41 (3), 187–190. 10.1111/j.1365-2125.1996.tb00181.x 8866917

[B5] Dehbozorgi M. Kamalidehghan B. Hosseini I. Dehghanfard Z. Sangtarash M. H. Firoozi M. (2018). Prevalence of the CYP2C19*2 (681 G>A), *3 (636 G>A) and *17 (-806 C>T) alleles among an Iranian population of different ethnicities. Mol. Med. Rep. 17 (3), 4195–4202. 10.3892/mmr.2018.8377 29328413 PMC5802190

[B6] Floren L. C. Bekersky I. Benet L. Z. Mekki Q. Dressler D. Lee J. W. (1997). Tacrolimus oral bioavailability doubles with coadministration of ketoconazole. Clin. Pharmacol. Ther. 62 (1), 41–49. 10.1016/S0009-9236(97)90150-8 9246018

[B7] Franco A. Mas-Serrano P. Balibrea N. Rodriguez D. Javaloyes A. Diaz M. (2019). Envarsus, a novelty for transplant nephrologists: observational retrospective study. Nefrol. Engl. Ed. 39 (5), 506–512. 10.1016/j.nefro.2018.11.009 30850218

[B8] Harvey A. T. Preskorn S. H. (2001). Fluoxetine pharmacokinetics and effect on CYP2C19 in young and elderly volunteers. J. Clin. Psychopharmacol. 21 (2), 161–166. 10.1097/00004714-200104000-00007 11270912

[B9] Hosohata K. Masuda S. Katsura T. Takada Y. Kaido T. Ogura Y. (2009). Impact of intestinal CYP2C19 genotypes on the interaction between tacrolimus and omeprazole, but not lansoprazole, in adult living-donor liver transplant patients. Drug Metab. Dispos. 37 (4), 821–826. 10.1124/dmd.108.025833 19139162

[B10] Itagaki F. Homma M. Yuzawa K. Fukao K. Kohda Y. (2002). Drug interaction of tacrolimus and proton pump inhibitors in renal transplant recipients with CYP2C19 gene mutation. Transpl. Proc. 34 (7), 2777–2778. 10.1016/s0041-1345(02)03409-7 12431607

[B11] Jeong S. Nguyen P. D. Desta Z. (2009). Comprehensive *in vitro* analysis of voriconazole inhibition of eight cytochrome P450 (CYP) enzymes: major effect on CYPs 2B6, 2C9, 2C19, and 3A. Antimicrob. Agents Chemother. 53 (2), 541–551. 10.1128/AAC.01123-08 19029318 PMC2630638

[B12] Maguire M. Franz T. Hains D. S. (2012). A clinically significant interaction between tacrolimus and multiple proton pump inhibitors in a kidney transplant recipient. Pediatr. Transpl. 16 (6), E217–E220. 10.1111/j.1399-3046.2011.01559.x 21883747

[B13] Marfo K. Altshuler J. Lu A. (2010). Tacrolimus pharmacokinetic and pharmacogenomic differences between adults and pediatric solid organ transplant recipients. Pharmaceutics 2 (3), 291–299. 10.3390/pharmaceutics2030291 27721357 PMC3967138

[B14] Mei S. Wang J. Chen D. Zhu L. Zhao M. Tian X. (2018). Simultaneous determination of cyclosporine and tacrolimus in human whole blood by ultra-high performance liquid chromatography tandem mass spectrometry and comparison with a chemiluminescence microparticle immunoassay. J. Chromatogr. B Anal. Technol. Biomed. Life Sci. 1087, 36–42. 10.1016/j.jchromb.2018.04.028 29704799

[B15] Miedziaszczyk M. Idasiak-Piechocka I. (2023). Safety analysis of co-administering tacrolimus and omeprazole in renal transplant recipients - a review. Biomed. Pharmacother. 166, 115149. 10.1016/j.biopha.2023.115149 37619481

[B16] Moreau C. Debray D. Loriot M. A. Taburet A. M. Furlan V. (2006). Interaction between tacrolimus and omeprazole in a pediatric liver transplant recipient. Transplantation 81 (3), 487–488. 10.1097/01.tp.0000194861.59543.b9 16477241

[B17] National Center for Biotechnology Information PubChem compound summary for CID 445643, tacrolimus.2005 Access: https://pubchem.ncbi.nlm.nih.gov/compound/Tacrolimus .

[B18] National Center for Biotechnology Information 2005 PubChem compound summary for CID 4594, omeprazole. Access: https://pubchem.ncbi.nlm.nih.gov/compound/Omeprazole .

[B19] National Center for Biotechnology Information PubChem compound summary for CID 5702160, famotidine. 2005 Access: https://pubchem.ncbi.nlm.nih.gov/compound/Amfamox .

[B20] Pascual J. Marcen R. Orea O. E. Navarro M. Alarcon M. C. Ocana J. (2005). Interaction between omeprazole and tacrolimus in renal allograft recipients: a clinical-analytical study. Transpl. Proc. 37 (9), 3752–3753. 10.1016/j.transproceed.2005.09.126 16386527

[B21] Pauli-Magnus C. Rekersbrink S. Klotz U. Fromm M. F. (2001). Interaction of omeprazole, lansoprazole and pantoprazole with P-glycoprotein. Naunyn Schmiedeb. Arch. Pharmacol. 364 (6), 551–557. 10.1007/s00210-001-0489-7 11770010

[B22] Peloso L. J. Faria P. N. Bossolani M. V. de Oliveira H. B. Ferreira Filho S. R. (2014). The serum concentration of tacrolimus after ingesting omeprazole: a pilot study. Transplantation 98 (6), e63–e64. 10.1097/TP.0000000000000351 25221905

[B23] Peng W. Lin Y. Zhang H. Meng K. (2020). Effect of ABCB1 3435C>T genetic polymorphism on pharmacokinetic variables of tacrolimus in adult renal transplant recipients: a systematic review and meta-analysis. Clin. Ther. 42 (10), 2049–2065. 10.1016/j.clinthera.2020.07.016 32888708

[B24] Ponticelli C. Passerini P. (2005). Gastrointestinal complications in renal transplant recipients. Transpl. Int. 18 (6), 643–650. 10.1111/j.1432-2277.2005.00134.x 15910287

[B25] Sugimoto M. Furuta T. (2012). Efficacy of esomeprazole in treating acid-related diseases in Japanese populations. Clin. Exp. Gastroenterol. 5, 49–59. 10.2147/CEG.S23926 22649281 PMC3359912

[B26] Summary of product characteristics of omeprazole Access: https://www.medicines.org.uk/emc/product/4895/smpc#gref ].

[B27] Summary of product characteristics of tacrolimus Access: https://www.medicines.org.uk/emc/product/7804/smpc ] (Accessed March 6, 2024).

[B28] Susal C. Dohler B. (2019). Late intra-patient tacrolimus trough level variability as a major problem in kidney transplantation: a Collaborative Transplant Study Report. Am. J. Transpl. 19 (10), 2805–2813. 10.1111/ajt.15346 30859672

[B29] Takahashi K. Yano I. Fukuhara Y. Katsura T. Takahashi T. Ito N. (2007). Distinct effects of omeprazole and rabeprazole on the tacrolimus blood concentration in a kidney transplant recipient. Drug Metab. Pharmacokinet. 22 (6), 441–444. 10.2133/dmpk.22.441 18159131

[B30] Telkes G. Peter A. Tulassay Z. Asderakis A. (2011). High frequency of ulcers, not associated with *Helicobacter pylori*, in the stomach in the first year after kidney transplantation. Nephrol. Dial. Transpl. 26 (2), 727–732. 10.1093/ndt/gfq401 20603242

[B31] Tholking G. Fortmann C. Koch R. Gerth H. U. Pabst D. Pavenstadt H. (2014). The tacrolimus metabolism rate influences renal function after kidney transplantation. PLoS One 9 (10), e111128. 10.1371/journal.pone.0111128 25340655 PMC4207775

[B32] Yasui-Furukori N. Takahata T. Nakagami T. Yoshiya G. Inoue Y. Kaneko S. (2004). Different inhibitory effect of fluvoxamine on omeprazole metabolism between CYP2C19 genotypes. Br. J. Clin. Pharmacol. 57 (4), 487–494. 10.1111/j.1365-2125.2003.02047.x 15025747 PMC1884483

[B33] Zhao W. Fakhoury M. Maisin A. Baudouin V. Storme T. Deschenes G. (2012b). Pharmacogenetic determinant of the drug interaction between tacrolimus and omeprazole. Ther. Drug Monit. 34 (6), 739–741. 10.1097/FTD.0b013e318271b6e6 23042259

[B34] Zhao W. F. M. Maisin A. Baudouin V. Storme T. Deschenes G. Jacqz-Aigrain E. (2012a). Pharmacogenetic determinant of the drug interaction between tacrolimus and omeprazole. Ther. Drug Monit. 34 (6), 739–741. 10.1097/FTD.0b013e318271b6e6 23042259

