# Supplementary material for: Assessment of omeprazole and famotidine effects on the pharmacokinetics of tacrolimus in patients following kidney transplant–randomized controlled trial
Source: Front Pharmacol. 2024 Apr 4;15:1352323. doi: 10.3389/fphar.2024.1352323 (PMC11024357; doi:10.3389/fphar.2024.1352323)

Assessment of omeprazole and famotidine effects on the pharmacokinetics of tacrolimus in patients following kidney transplant - prospective cohort study

p.o. tacrolimus, mycophenolate mofetil, prednisone/methylprednisolone/deflazacort

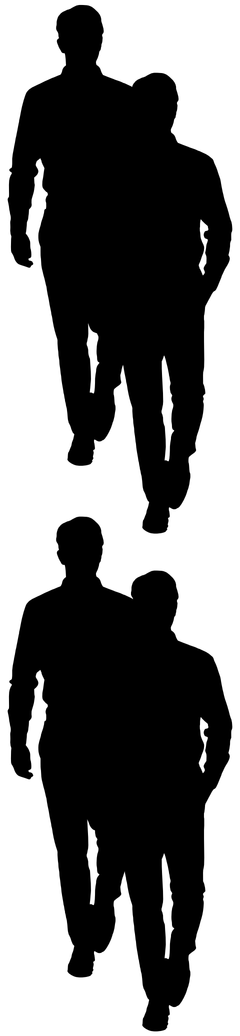

OM

omeprazole 20 mg

FA

famotidine 20 mg

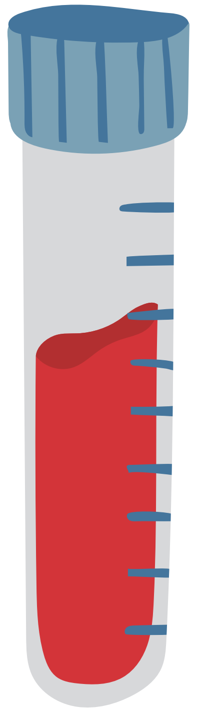

1 day 0 2h 6h 12h

2 day 0 2h 6h

CMIA

Chemiluminescent Microparticle Immuno Assay method

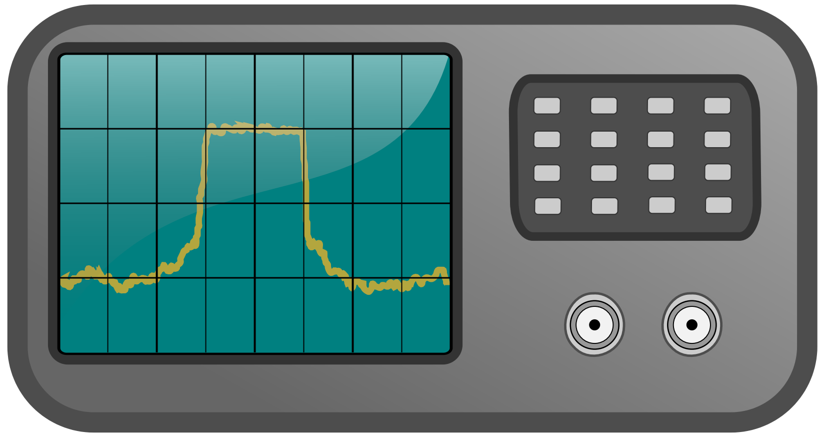

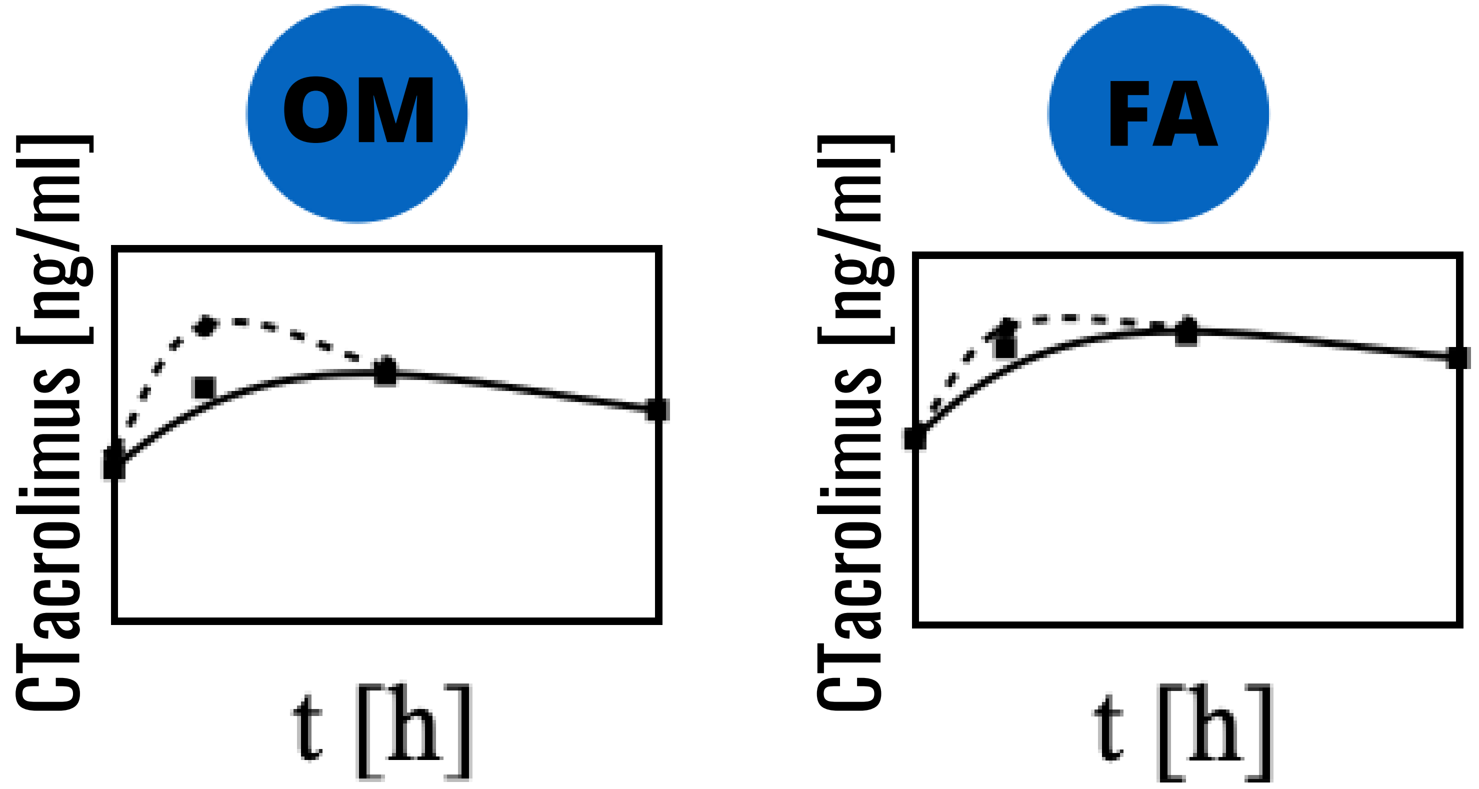

Supplement: Supplementary file 1 [file DataSheet2.PDF]
